# Attenuation of muscle atrophy in a murine model of cachexia by inhibition of the dsRNA-dependent protein kinase

**DOI:** 10.1038/sj.bjc.6603704

**Published:** 2007-03-27

**Authors:** H L Eley, S T Russell, M J Tisdale

**Affiliations:** 1 Nutritional Biomedicine, School of Life and Health Sciences, Aston University Biomedical Science, Birmingham B4 7ET, UK

**Keywords:** dsRNA-dependent protein kinase, inhibition, muscle protein synthesis, muscle protein degradation, cancer cachexia

## Abstract

Atrophy of skeletal muscle is due to a depression in protein synthesis and an increase in degradation. Studies *in vitro* have suggested that activation of the dsRNA-dependent protein kinase (PKR) may be responsible for these changes in protein synthesis and degradation. In order to evaluate whether this is also applicable to cancer cachexia the action of a PKR inhibitor on the development of cachexia has been studied in mice bearing the MAC16 tumour. Treatment of animals with the PKR inhibitor (5 mg kg^−1^) significantly reduced levels of phospho-PKR in muscle down to that found in non-tumour-bearing mice, and effectively attenuated the depression of body weight, with increased muscle mass, and also inhibited tumour growth. There was an increase in protein synthesis in skeletal muscle, which paralleled a decrease in eukaryotic initiation factor 2*α* phosphorylation. Protein degradation rates in skeletal muscle were also significantly decreased, as was proteasome activity levels and expression. Myosin levels were increased up to values found in non-tumour-bearing animals. Proteasome expression correlated with a decreased nuclear accumulation of nuclear factor-*κ*B (NF-*κ*B). The PKR inhibitor also significantly inhibited tumour growth, although this appeared to be a separate event from the effect on muscle wasting. These results suggest that inhibition of the autophosphorylation of PKR may represent an appropriate target for the attenuation of muscle atrophy in cancer cachexia.

Cancer cachexia is characterised by specific depletion of skeletal muscle mass, which leads to a general muscle weakness (asthenia) and reduced physical activity, terminating in impairment of respiratory muscle function and death of the patient through hypostatic pneumonia (Windsor and Hill, 1988). In order to counteract this progressive muscle atrophy, it is necessary to understand the mechanisms involved. Protein loss from skeletal muscle results from a combination of a depression in protein synthesis (Emery *et al*, 1984), together with an increase in protein degradation (Lundholm *et al*, 1982), which is initiated by cachectic factors. We have recently shown a relationship between the depression of protein synthesis in skeletal muscle and the increase in protein degradation in response to cachectic factors such as proteolysis-inducing factor (PIF) and angiotensin II (Ang II) though the dsRNA-dependent protein kinase (PKR) (Eley and Tisdale, 2007). Thus, using murine myotubes *in vitro* both agonists were found to induce autophosphorylation and activation of PKR. One of the primary substrates for PKR is eukaryotic initiation factor 2 (eIF2), which is phosphorylated on the *α*-subunit, leading to inhibition of translation initiation by blocking the action of the guanine exchange factor eIF2B (Panniers and Henshaw, 1983). Both PIF and Ang II inhibited protein synthesis in myotubes, and this was attenuated in myotubes transfected with a catalytically inactive variant of PKR (PKRΔ6), which showed no increase in eIF2*α* phosphorylation in response to PIF and Ang II. Inhibition of PKR by a low molecular weight inhibitor also attenuated the depression of protein synthesis, confirming that it arose from an increased phosphorylation of eIF2*α* (Eley and Tisdale, 2007).

In addition, PKR has been shown to mediate activation of the transcription factor, nuclear factor-*κ*B (NF-*κ*B) through activation of the upstream kinase, I*κ*B kinase leading to degradation of the inhibitors I*κ*B*α* and I*κ*B*β* and the concomitant release of NF-*κ*B (Zamanian-Daryoush *et al*, 2000). Activation of NF-*κ*B has been shown to cause muscle atrophy due to accelerated protein breakdown through increased expression of the key components of the ubiquitin–proteasome proteolytic pathway including proteasome subunits and the E3 ligase, MuRF1 (Cai *et al*, 2004). Induction of the ubiquitin–proteasome pathway by both PIF (Wyke and Tisdale, 2005) and Ang II (Russell *et al*, 2006a, 2006b) also requires activation of NF-*κ*B. Myotubes treated with a PKR inhibitor, or containing plasmids expressing mutant PKRΔ6, showed no protein degradation in response to PIF or Ang II, and no activation of NF-*κ*B, confirming a link between activation of PKR and the induction of protein degradation, as well as the depression of protein synthesis in skeletal muscle (Eley and Tisdale, 2007).

If this same process is operative in skeletal muscle during cancer cachexia then inhibitors of PKR may be useful therapeutically to prevent muscle atrophy. Certainly phosphorylation of both PKR and eIF2*α* was found to be increased in the gastrocnemius muscle of weight losing mice bearing the MAC16 tumour (Eley and Tisdale, 2007). To test the hypothesis that inhibition of PKR may prevent muscle atrophy, the present study investigates the effect of a small molecule ATP-site directed inhibitor of PKR, 8-[1-(1H-imidazol-4-yl) meth-(Z) ylidene]-6,8-dihydro-thiazol [5,4-e]indol-7-one (Jammi *et al*, 2003), on cachexia in the MAC16 model. It would be anticipated that inhibition of PKR would attenuate the depression of protein synthesis in skeletal muscle, through a reduction in the phosphorylation state of eIF2*α*, and would also attenuate the increased protein degradation, by downregulating the expression of the ubiquitin–proteasome pathway, by preventing the activation of NF-*κ*B.

## MATERIALS AND METHODS

### Materials

L-[2, 6-^3^H] Phenylalanine (sp. act.2.07TBq mmol^−1^), hybond A nitrocellulose membranes and enhanced chemiluminescene (ECL) development kits were from Amersham Biosciences Ltd (Bucks, UK). Mouse monoclonal antibodies to 20S proteasome *α*-subunits and p42 were from Affiniti Research Products (Exeter, UK). Rabbit monoclonal antibodies to phospho-eIF2*α* (Ser 51) and to phospho-PKR (Thr 446) were purchased from Insight Biotechnology Ltd (London, UK). Mouse monoclonal antibody to myosin heavy chain was from Novocastra (Newcastle, UK), whereas polyclonal antisera to total PKR were from New England Biolabs Ltd (Herts, UK). Rabbit polyclonal antisera to mouse *β*-actin were from Sigma Aldridge (Dorset, UK). Peroxidase-conjugated goat anti-rabbit antibody and peroxidase-conjugated rabbit anti-mouse antibody were purchased from Dako Ltd (Cambridge, UK). The PKR inhibitor and PhosphoSafe™ Extraction Reagent were from Merck Eurolab Ltd (Leicestershire, UK) and electrophoretic mobility shift assay (EMSA) gel shift assay kits were from Panomics (CA, USA).

### Animals

Pure strain male NMRI mice (average weight 25 g) were obtained from our own inbred colony and were fed a rat and mouse breeding diet (Special Diet Services, Witham, UK) and water *ad libitum*. Animals were transplanted with fragments of the MAC16 tumour s.c. into the flank by means of a trochar, as described (Bibby *et al*, 1987), selecting from donor animals with established weight loss. Weight loss was evident 12–15 days after tumour transplantation and animals were entered into the study when they had lost approximately 5% of their starting body weight. Animals were randomised into groups of six to receive solvent (DMSO PBS; 1 : 20) or the PKR inhibitor (at 1 and 5 mg kg^−1^) administered daily by s.c. injection. Both tumour volume and body weight were monitored daily. Animals were terminated by cervical dislocation when the body weight loss reached 25%, and all animal experiments followed a strict protocol approved by the British Home Office, and the ethical guidelines that were followed meet the standards required by the UKCCR guidelines (Workman *et al*, 1998). The soleus muscles were quickly dissected out, together with intact tendons maintained in isotonic ice-cold saline before determination of protein synthesis. Protein degradation was determined on freshly excised gastrocnemius muscle.

### Protein synthesis and degradation in muscle

The method for the determination of protein synthesis in muscle has been previously described (Smith and Tisdale, 1993). Protein synthesis was measured by the incorporation of L-[2, 6-^3^H] phenylalanine into acid insoluble material during a 2 h period in which soleus muscles were incubated at 37°C in RPMI 1640 without phenol red and saturated with O_2_ : CO_2_ (19 : 1). After incubation, muscles were rinsed in non-radioactive medium, blotted and homogenised in 4 ml 2% perchloric acid. The rate of protein synthesis was calculated by dividing the amount of protein-bound radioactivity by the amount of acid-soluble radioactivity.

For protein degradation, gastrocnemius muscle was incubated in 3 ml of oxygenated (95% oxygen; 5% carbon dioxide) Krebs–Henselit buffer (pH 7.4), containing 5 mM glucose and 0.5 mM cycloheximide. The protein degradation rate was determined by the release of tyrosine (Waalkes and Udenfriend, 1957) over a 2 h period.

### Measurement of proteasome activity

The 20S proteasome functional activity was determined by measuring the ‘chymotrypsin-like’ activity by the method of Orino *et al* (1991), which measures the release of aminomethyl coumarin (AMC) from the fluorogenic peptide succinyl-LLVY-AMC in the absence and presence of the specific proteasome inhibitor lactacystin (10 *μ*M). Only lactacystin-suppressible activity was considered to be proteasome specific. Activity was normalised to the protein content of the muscle determined by the Bradford assay (Sigma).

### Body composition analysis

After killing animals were heated to 80–90°C for 48 h, or until a constant weight was achieved. The water content was determined from the difference between the wet and dry weight. Lipids were extracted from the dried carcass with chloroform/methanol (1 : 1), ethanol/acetone (1 : 1) and diethyl ether, which was then allowed to evaporate. The fat content was determined from the weighed residue. The non-fat carcass mass was calculated as the difference between the initial weight of the carcass and the weight of water and fat.

### Electrophoretic mobility shift assay

DNA-binding proteins were extracted from skeletal muscle using hypotonic lysis followed by high salt extraction of nuclei, as described (Andrews and Faller, 1991). The EMSA-binding assay was carried out using a Panomics EMSA ‘gel shift’ kit according to the manufacturer's instructions.

### Western blot analysis

Samples (about 10 mg) of gastrocnemius muscle were homogenised in 500 *μ*l PhosphoSafe™ Extraction Reagent and centrifuged at 18 000 **g** for 5 min. Samples of cytosolic protein (10 *μ*g) were resolved on 10% sodium dodecylsulphate–polyacrylamide gel electrophoresis (6% for eIF2*α*), and transferred to 0.45 *μ*m nitrocellulose membranes, which had been blocked with 5% Marvel in Tris-buffered saline, pH 7.5, at 4°C for 1–2 h, and then washed for 15 min in 0.1% Tween buffered saline or PBS Tween before adding the primary antibodies. The primary antibodies were used at a dilution of 1 : 1000, except for phospho-eIF2*α* (1 : 500), actin (1 : 200) and myosin (1 : 100). The secondary antibodies were used at a dilution of 1 : 1000. Incubation was for 1 h at room temperature (actin, p42) or overnight, and development was by ECL. Blots were scanned by a densitometer to quantify differences.

### Statistical analysis

Results are presented as mean±s.e.m. Differences in means between groups were determined by one-way analysis of variance followed by Tukey–Kramer multiple comparison test. *P*-values less than 0.05 were considered significant.

## RESULTS

**Figure 1:**
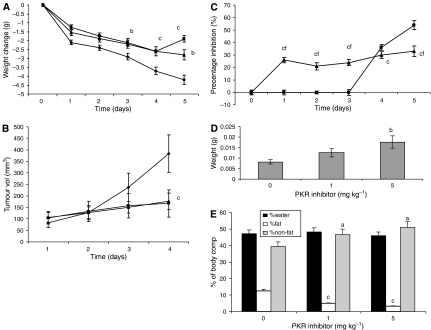
Effect of daily s.c. administration of a PKR inhibitor at 1 (▪) and 5 (▴) mg kg^−1^ in comparison with solvent control (DMSO : PBS 1 : 20) on body weight change (**A**) and tumour growth rate (**B**) in mice bearing the MAC16 tumour. A time course for the inhibition of body weight loss (▴) and tumour growth (▪) is shown in (**C**). The average weight of the soleus muscles after 5 days treatment is shown in (**D**), and the body composition is shown in (**E**). The conditions for tumour transplantation and conductance of the experiment are given in Materials and Methods. The number of mice in each group *n*=6. Differences from control are shown as a: *P*<0.05; b: *P*<0.01; or c: *P*<0.001, whereas differences from percentage inhibition of tumour volume are shown as f: *P*<0.001.

**Figure 2:**
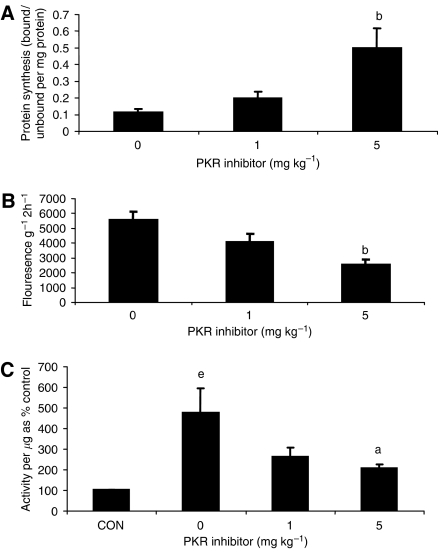
Protein synthesis (**A**), protein degradation (**B**) and ‘chymotrypsin-like’ enzyme activity, in comparison with a non-tumour-bearing control (**C**) in the skeletal muscle of mice bearing the MAC16 tumour after 5 days of treatment, as shown in Figure 1. The number of muscles used in each group *n*=6. Differences from animals not receiving inhibitor are shown as a: *P*<0.05 or b: *P*<0.01, whereas differences from non-tumour-bearing animals is shown as e: *P*<0.01.

**Figure 3:**
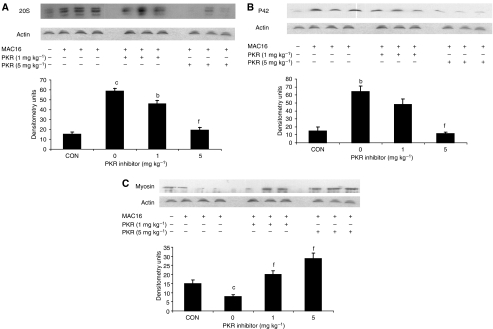
Western blots of 20S proteasome *α*-subunits (**A**), p42 (**B**) and myosin (**C**) in gastrocnemius muscle of mice bearing the MAC16 tumour after 5 days treatment with a PKR inhibitor (1 and 5 mg kg^−1^), as described in the legend to Figure 1 in comparison with values from non-tumour-bearing animal (NTB). Actin was used as a loading control. The first lane (CON) used gastrocnemius muscle from a non-tumour-bearing control. Representative blots are shown and the densitometric analysis is the average of at least three separate blots. Differences from NTB control are shown as b: *P*<0.01 or c: *P*<0.001, whereas differences from the solvent control are indicated as f: *P*<0.001 (*n*=9).

**Figure 4:**
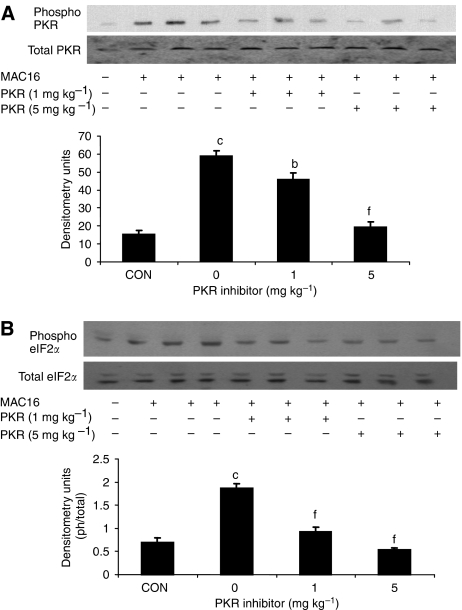
Western blots of phospho-PKR (**A**) and -eIF2*α* (**B**) in gastrocnemius muscle of mice bearing the MAC16 tumour after 5 days treatment with a PKR inhibitor (1 and 5 mg kg^−1^), as described in the legend to Figure 1. The blots for total PKR and eIF2*α* were used as loading controls. The first lane (CON) used gastrocnemius muscle from an NTB control. Representative blots are shown and the densitometric analysis gives the ratio of the phospho to total forms as an average of three separate blots (*n*=9). Differences from NTB control are shown as a: *P*<0.05 or c: *P*<0.001, whereas differences from the solvent control are indicated as f: *P*<0.01.

**Figure 5:**
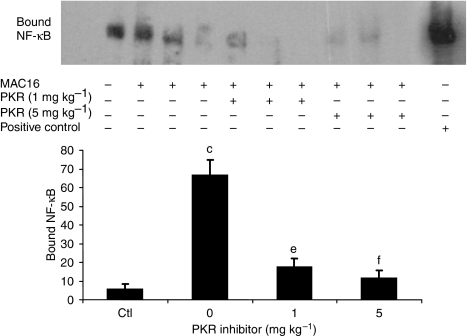
(**A**) EMSA of nuclear binding of NF-*κ*B in gastrocnemius muscle of non-tumour-bearing mice (lane 1) or mice bearing the MAC16 tumour and treated with solvent alone (lanes 2–4) or the PKR inhibitor at 1 (lanes 5–7) or 5 mg kg^−1^ (lanes 8–10). Lane 11 is a positive control for NF-*κ*B (supplied by the manufacturer of the kit). (**B**) Densitometric analysis of the EMSA shown in (**A**), *n*=3. Differences from non-tumour-bearing controls are shown as c: *P*<0.001, whereas differences from solvent control are shown as e: *P*<0.01 or f: *P*<0.001.

The effect of the PKR inhibitor at two dose levels (1 and 5 mg kg^−1^) on change in body weight and tumour growth in mice bearing the MAC16 tumour over a 5-day period is shown in Figure 1. The dose levels were chosen based on the effective dose *in vitro* (Eley and Tisdale, 2007), and by using dose-range finding assays to determine toxicity. At dose levels of both 1 and 5 mg kg^−1^, the PKR inhibitor effectively attenuated both the depression in body weight (Figure 1A) and tumour growth (Figure 1B), although the time course for these two events appeared to be distinct (Figure 1C). In addition, in animals treated with the PKR inhibitor at 5 mg kg^−1^ there was a significant increase in muscle wet weight suggesting preservation of muscle mass (Figure 1D), and this was confirmed by body composition analysis (Figure 1E), which showed a significant increase in the non-fat carcass mass. Body composition analysis also showed that at both dose levels of the PKR inhibitor there was a significant depression in the carcass fat mass. At this dose level, there was a significant increase in protein synthesis in skeletal muscle (Figure 2A), which paralleled the decrease in eIF2*α* phosphorylation (4B). There was also a significant decrease in protein degradation (Figure 2B). The latter was reflected in a significant decrease in the functional activity of the 20S proteasome, as measured by the ‘chymotrypsin-like’ enzyme activity (Figure 2C), such that at a dose level of 5 mg kg^−1^enzyme levels were reduced down to that found in non-tumour-bearing controls. In addition, expression of the 20S proteasome *α*-subunits (Figure 3A), and p42, an ATPase subunit of the 19S regulator in skeletal muscle (Figure 3B), were reduced down to the levels found in non-tumour-bearing mice. Also mice bearing the MAC16 tumour showed a significant depression in the expression of the myofibrillar protein myosin, and this was restored up to the levels found in non-tumour-bearing animals after treatment with both dose levels of the PKR inhibitor (Figure 3C). As previously reported (Eley and Tisdale, 2007), levels of both phospho-PKR (Figure 4A) and -eIF2*α* (Figure 4B) were significantly increased in the skeletal muscle of mice bearing the MAC16 tumour, and this was reduced down to levels found in non-tumour-bearing animals after treatment with the PKR inhibitor. To verify that changes in proteasome expression in skeletal muscle arose from an effect on nuclear migration of NF-*κ*B, the amount of NF-*κ*B in the nucleus was determined by EMSA. The results depicted in Figure 5 show a significant upregulation of NF-*κ*B in gastrocnemius muscle of mice bearing the MAC16 tumour, in comparison with non-tumour-bearing control, and a reduction in NF-*κ*B DNA-binding activity in gastrocnemius muscle of mice treated with the PKR inhibitor at both dose levels, down to values found in non-tumour-bearing controls. These results suggest that inhibition of PKR autophosphorylation may be a useful target in attenuating muscle atrophy in cancer cachexia.

## DISCUSSION

PKR is a serine/threonine protein kinase, which is normally inactive, but undergoes a conformational change upon binding of its activator, dsRNA, leading to autophosphorylation, with phosphorylation of substrates independent of dsRNA (Clemens, 1997). The best characterised substrate of PKR is the *α*-subunit of eIF2, which results in the sequestration of the recycling factor eIF2B in an inactive complex with eIF2-GDP, inhibiting protein synthesis (Panniers and Henshaw, 1983). Studies *in vitro* using PIF and Ang II as agonists (Eley and Tisdale, 2007) have shown that activation of PKR not only depresses protein synthesis but also increases protein degradation through an NF-*κ*B-dependent increase in proteasome expression. Weight losing mice bearing the MAC16 tumour showed a similar increase in phospho-PKR and -eIF2*α*, in gastrocnemius muscle, suggesting that a similar mechanism was operative in cancer cachexia. We have recently shown (Eley and Tisdale, unpublished results) that levels of phospho-PKR and -eIF2*α* were also significantly elevated in rectus abdominis muscle of weight losing cancer patients, when the weight loss exceeded 10%. There was a parabolic relationship between levels of phospho-PKR and -eIF2*α* and weight loss, with levels increasing with weight loss up to a maximum of 19–20%, and then decreasing. There was a linear relationship between expression of mRNA for the C2 proteasome subunit and levels of phospho-PKR, suggesting that phosphorylation of PKR was responsible for the induction of proteasome expression and degradation of myofibrillar proteins. Thus, if the *in vitro* results are also applicable *in vivo* this suggests that inhibition of PKR autophosphorylation may be useful for the treatment of muscle atrophy in cancer patients, particularly for those with weight losses between 10 and 20%.

**Figure 6:**
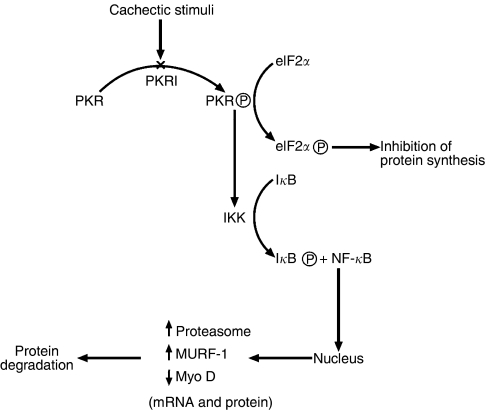
Flow diagram showing how activation of PKR leads to inhibition of protein synthesis through phosphorylation of eIF2*α*; and increased protein degradation through activation of NF-*κ*B, which would be attenuated by a PKR inhibitor (PKRI).

To test this hypothesis, the current study examined the effect of a PKR inhibitor on muscle wasting in mice bearing the cachexia-inducing MAC16 tumour. The results of this study confirm that inhibition of PKR phosphorylation, by a small molecule inhibitor, attenuates the development of cachexia in a murine model, through an increase in non-fat carcass mass, although the effect on total body weight is less pronounced because of a significant depression of the carcass fat mass. The mechanism for this effect is not known. The PKR inhibitor preserved muscle mass through the attenuation of the depression of protein synthesis and the increase in protein degradation in skeletal muscle, as predicted from the *in vitro* study (Eley and Tisdale, 2007). Inhibition of PKR activity was found to attenuate phosphorylation of eIF2*α*, which would prevent the depression of protein synthesis through inhibition of translation initiation. As with the *in vitro* model (Eley and Tisdale, 2007), inhibition of the phosphorylation of PKR also attenuated the increased protein degradation in the skeletal muscle of cachectic mice through repression of the induction of proteasome expression and activity concomitant with a significantly reduced nuclear accumulation of NF-*κ*B down to values found in non-tumour-bearing animals. The increased nuclear accumulation of NF-*κ*B in cachectic mice bearing the MAC16 tumour is probably due to tumour factors such as PIF, which has been shown to induce expression of the ubiquitin–proteasome system through activation of NF-*κ*B (Wyke and Tisdale, 2005). Decreased activation of NF-*κ*B would also be expected to reduce expression of the E3 ligase, MuRF1 (Cai *et al*, 2004), and to increase levels of the transcription factor MyoD (Guttridge *et al*, 2000), which is essential for skeletal muscle differentiation, and for repair of damaged tissue. MyoD expression has been shown to be dramatically downregulated in skeletal muscle of cachectic rats (Costelli *et al*, 2005). Both of these changes would contribute to increased levels of the myofibrillar protein, myosin, in skeletal muscle. A flow diagram showing the proposed pathways by which a PKR inhibitor could attenuate the depression of protein synthesis and increase protein degradation in skeletal muscle is shown in Figure 6. In addition to this pathway, there is an NF-*κ*B-independent pathway involved in ubiquitin-mediated proteolysis involving the transcription factor Foxo1 and which is activated by myostatin (McFarlane *et al*, 2006). There is no evidence that this pathway would be affected by inhibition of PKR.

A surprising observation was that inhibition of PKR phosphorylation also inhibited tumour growth. This was unlikely to be responsible for the observed changes in protein synthesis and degradation in skeletal muscle, as it occurred after the stabilisation of body weight (Figure 1C). Thus, the PKR inhibitor had a rapid effect on the loss of body weight, which was apparent within 1 day of treatment and remained at the same level throughout the experiment. In contrast, inhibition of tumour growth was not evident until day 4 of the experiment, and there was a progressive increase between days 4 and 5. dsRNA-dependent protein kinase has been suggested to act as a tumour suppressor protein (Clemens, 1997), as it probably functions in interferon-mediated host defence to trigger cell death in response to viral infection and possible tumorigenesis (Balachandran *et al*, 1998). Overexpression of PKR in mammalian and insect cells results in inhibition of cellular growth, which probably involves repression of translation through inhibition of the eIF2*α* pathway (Barber, 2005), whereas the expression of catalytically inactive dominant-negative PKR molecules causes the transformation of immortalised cells (Koromilas *et al*, 1992). Thus, inhibition of PKR phosphorylation might be expected to stimulate tumour growth. However, PKR can also mediate activation of NF-*κ*B (Zamanian-Daryoush *et al*, 2000), although it has not been shown that this happens in tumours. Nuclear factor-*κ*B is known to be constitutively activated in certain tumours, including the MAC16 tumour (Wyke *et al*, 2004), and this has been connected with tumour cell survival and proliferation, as well as invasion and angiogenesis (Baldwin, 2001; Karin *et al*, 2002). Thus, inhibition of PKR phosphorylation might be expected to downregulate nuclear binding of NF-*κ*B, as it does in skeletal muscle.

Another possible explanation is that the MAC16 tumour requires amino acids released from skeletal muscle during proteolysis for growth. We have found (Hussey and Tisdale, unpublished results) that MAC16 cells have a higher requirement for isoleucine and tryptophan than a related tumour (MAC13), which does not induce cachexia. Previous studies have shown inhibition of the growth of the MAC16 tumour when cachexia was attenuated by *β*-hydroxy-*β*-methylbutyrate, a metabolite of leucine (Smith *et al*, 2005) and resveratrol at low dose levels (Wyke *et al*, 2004).

More studies are needed on the antitumour activity of PKR inhibitors and the mechanism by which they exert their effect. Inhibitors of PKR may also be expected to be synergistic with DNA-damaging agents such as cisplatin and melphalan, as PKR-deficient mouse-embryonic fibroblasts have been shown to be hypersensitive to bulky adduct DNA damage (Bergeron *et al*, 2000).

These results suggest that agents which inhibit autophosphorylation of PKR may be useful in the treatment of muscle atrophy associated with cancer cachexia and may have the added benefit of attenuating tumour growth.
